# LPHN3, a presynaptic adhesion-GPCR implicated in ADHD, regulates the strength of neocortical layer 2/3 synaptic input to layer 5

**DOI:** 10.1186/1749-8104-9-7

**Published:** 2014-04-17

**Authors:** Matthew L O’Sullivan, Francesca Martini, Sventja von Daake, Davide Comoletti, Anirvan Ghosh

**Affiliations:** 1 Neurobiology Section, Division of Biology, University of California San Diego, La Jolla, CA 92093, USA; 2 Current affiliation: Duke University School of Medicine, Durham, NC 27710, USA; 3 Child Health Institute of New Jersey and Department of Neuroscience and Cell Biology, Robert Wood Johnson Medical School, Rutgers University, New Brunswick, NJ 08901, USA; 4 Neuroscience Discovery, F. Hoffmann-LaRoche, 4070 Basel, Switzerland

## Abstract

**Background:**

Latrophilins (LPHNs) are a small family of neuronal adhesion-GPCRs originally discovered as receptors for the black widow spider toxin α-latrotoxin. Mutations in *LPHN3* have recently been identified as risk factors for attention deficit hyperactivity disorder (ADHD) in humans, but their physiological function has remained elusive. In this study, we tested two hypotheses regarding LPHN3 function: (1) LPHN3 regulates synaptic transmission by modulating probability of release; and (2) LPHN3 controls synapse development and the abundance of synapses.

**Results:**

We manipulated LPHN3 expression in mouse layer 2/3 (L2/3) pyramidal neurons and examined the consequences on the L2/3 to L5 cortical microcircuit. Employing an optogenetic strategy combined with shRNA knockdown of LPHN3, we found that LPHN3 did not influence probability of release at synapses formed by L2/3 neurons onto L5 pyramidal cells. The strength of L2/3 afferent input to L5, however, was weakened by loss of LPHN3. Using Synaptophysin-GFP as an anatomical marker of presynaptic terminals, we found that the density of synapses formed by L2/3 axons in L5 was reduced when LPHN3 was lost. Finally, we investigated the structural organization of the extracellular domain of LPHN3. We used single particle negative stain electron microscopy to image the extracellular domain of LPHN3 and showed that the Olfactomedin and Lectin domains form a globular domain on an elongated stalk. Cell-based binding experiments with mutant proteins revealed that the Olfactomedin domain was required for binding to FLRT3, whereas both the Olfactomedin and Lectin domains were involved in binding to Teneurin 1. Mutant LPHN3 lacking the Olfactomedin domain was not capable of rescuing the deficit in presynaptic density following knockdown of endogenous LPHN3.

**Conclusions:**

We find that LPHN3 regulates the number of synapses formed by L2/3 neurons in L5 and the strength of synaptic drive from the L2/3-L5 pathway. The Olfactomedin domain of LPHN3 is required for this effect on synapse number and binding to its postsynaptic ligand FLRT3. We propose that LPHN3 functions in synaptic development and is important in determining the connectivity rates between principal neurons in the cortex.

## Background

Latrophilins (LPHNs) are G protein-coupled receptors (GPCRs) with adhesion-like extracellular domains [1]. Recently, *LPHN3* has been linked to attention deficit hyperactivity disorder (ADHD); polymorphisms in LPHN3 are risk factors for ADHD [2,3] and LPHN3 haplotype can predict the efficacy of stimulant treatment [4] and human cortical electrophysiology [5]. This mounting genetic evidence suggests that LPHN3 may be important for cognition and cortical function.

Latrophilins were discovered due to their role as receptors for α-latrotoxin, a venom protein produced by spiders of the *Latrodectus* genus [1]. α-latrotoxin is a potent exocytotic agent whose effect on presynaptic terminals can be mediated by LPHN1, suggesting that LPHNs are functionally coupled to the presynaptic active zone [1]. Known presynaptic GPCRs are predominantly metabotropic neurotransmitter receptors that modulate synaptic transmission by influencing the function of the active zone and voltage-gated calcium channels [6]. The combination of α-latrotoxin toxicology and the precedent set by metabotropic neurotransmitter receptors has lead to speculation that LPHNs might also modulate synaptic transmission [7].

Unlike metabotropic receptors, however, the endogenous ligands for the LPHNs are transmembrane proteins (the FLRTs and Teneurins) rather than small diffusible molecules [7-9]. Consequently, the concentration of available ligand for LPHNs is likely to vary only slowly over time, potentially making LPHN signaling inappropriate for fast modulation of synaptic transmission. The function of LPHNs might then manifest at longer, developmental timescales. Indeed, we previously found that postsynaptic fibronectin leucine-rich transmembrane (FLRT) proteins regulate glutamatergic synapse density in hippocampal neurons *in vitro* and *in vivo*[8]. Their participation in trans-synaptic interactions may imply a role for LPHNs in regulating synaptic development.

While LPHN1 and LPHN3 are broadly expressed in the brain, there is evidence that FLRTs and Teneurins (TENs) show differential patterns of expression [8,10-13]. In the neocortex, *Flrt1* and *Flrt3* expression is highest in layer 2/3 (L2/3) and L5, while *Flrt2* seems to be more broadly expressed across layers [8,12,13]. Of the Teneurin genes, *Tenm1* shows the strongest expression, predominately in L5 and L6 [10-13]. The broad expression of LPHNs and more restricted expression of its FLRT and TEN ligands may further imply that LPHNs may interact with different ligands in different contexts.

Since α-latrotoxin induces synaptic vesicle release and *LPHN3* mutations are linked to cortical function through their association with ADHD, we chose to investigate two hypotheses regarding the role of presynaptic LPHN3 at cortical synapses: (1) LPHN3 modulates presynaptic vesicle release; and (2) LPHN3 regulates synapse development. We manipulated layer 2/3 (L2/3) pyramidal neurons in the mouse cortex *in vivo* by *in utero* electroporation, and examined the L2/3 to L5 microcircuit. We found that loss of LPHN3 reduced the strength of L2/3 input to L5 and the number of synapses formed by L2/3 axons, but that manipulating LPHN3 levels did not affect the probability of release at L2/3 terminals. We then investigated the structure of the LPHN3 extracellular domain and mapped the regions involved in interaction with its ligands, the FLRTs and Teneurins (TENs). Binding to both FLRTs and TENs depends on regions found within the distal globular domain, but we found that the Olfactomedin domain is required for interaction with FLRTs while both the Lectin and Olfactomedin domains are involved in the interaction with TENs. Using LPHN3 deletion mutants in a functional rescue experiment, we find that the Olfactomedin but not Lectin domain is required to support synapse development. Our data indicate that the architecture of the extracellular domain of LPHN3 contains a distal ‘ligand binding module’ with differentiable FLRT3 and TEN1 binding sites, situated on an elongated and potentially flexible stalk long enough to span the synaptic cleft. We propose that LPHN3 does not regulate presynaptic function, but rather interacts trans-synaptically with FLRTs during synapse development and contributes to determining the density of synaptic terminals formed by axons of cortical pyramidal neurons.

## Results

### LPHN3 regulates the strength of L2/3 input to L5

Since LPHN3 is broadly expressed in the cortex and FLRTs and TENs are expressed in L5, we decided to investigate the role of LPHN3 at synapses of the L2/3 to L5 pathway, an important canonical element of cortical circuitry [8,10-13]. To test whether LPHN3 affects synapses formed by the axons of L2/3 pyramidal neurons, we used *in utero* electroporation to express Channelrhodopsin 2 (ChR2) in L2/3 pyramidal neurons with or without an shRNA to knock down LPHN3 expression (shLPHN3). This shRNA has been previously described, and strongly and specifically knocks down LPHN3 [8]. This strategy afforded us several advantages compared to more conventional techniques: (1) while a stimulating electrode in the cortex would stimulate a mixed population of axons, optogenetic stimulation allows for the stimulation of a pure population of L2/3 axons; and (2) co-electroporation of Cre-dependent ChR2 guarantees that only axons in which LPHN3 has been knocked down are light-sensitive, giving selective stimulation of a sparse manipulated population of axons on a wildtype background.

**Figure 1:**
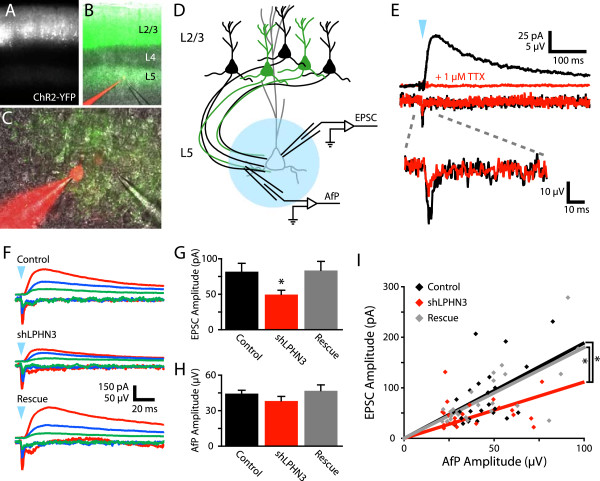
**LPHN3 regulates the strength of synaptic input from L2/3 to L5. (A-C)** Images of an acute coronal slice from a P14 mouse electroporated at E16 to express ChR2-YFP in L2/3 PNs. **(B, C)** A L5 PN is filled with Alexa 595 during a whole cell recording with a pipette nearby to record the axonal field potential (AfP). **(D)** Schematic of the optogenetic electrophysiology experiment. A whole cell recording from a L5 PN is used to record EPSCs while the AfP is recorded nearby. Flashes of blue light over the recorded cell (blue circle) are used to stimulated ChR2-expressing axon of L2/3 neurons in L5. **(E)** Example average EPSC (top: V_hold_ = +40 mV) and AfP (bottom) before (black) and after (red) 1 μM TTX. **(F)** Example recordings with small (green), medium (blue), and large (red) AfPs from different Control, shLPHN3, and Rescue slices. **(G)** shLPHN3 reduced mean EPSC amplitude versus Control and Rescue (one-way ANOVA, F(2,68) = 3.34, *P* = 0.04: Control 81.72 ± 11.42 pA, n = 25; shLPHN3 49.5 ± 5.8 pA, n = 23; Rescue 83.48 ± 12.42 pA, n = 23). **(H)** AfP amplitude did not differ (one-way ANOVA, F(2,68) = 1.06, *P* = 0.35: Control, 4.44 ± 0.27 pA, n = 25; shLPHN3, 3.89 ± 0.39 pA, n = 23; Rescue, 4.68 ± 0.48 pA, n = 23). **(I)** Plot of AfP *vs.* EPSC amplitude for Control (black), shLPHN3 (red), and Rescue (gray) inputs, fitted with a line through the origin. The slopes of the fitted lines differed between conditions (extra sum-of-squares F test, F(2,68) = 4.82, *P* = 0.01). The slope of the shLPHN3 condition (11.14 pA/μV, 95% confidence interval 7.82-14.46) was determined to be significantly different from Control (18.91, 14.69-23.12) and Rescue (17.97, 14.53-21.42) since the 95% confidence intervals were non-overlapping.

At 2 weeks of age, acute slices of somatosensory cortex were cut from electroporated mice. ChR2-expressing cell bodies in L2/3 and axons in L5 were clearly visible by YFP epifluorescence (Figure 1A,B). Recordings were made from L5 pyramidal neurons in regions where YFP-positive axons were abundant (Figure 1C). Slices were surveyed to ensure L5 neurons were not also expressing ChR2. Blue light flashes over L5 frequently evoked polysynaptic excitation under our conditions, so we decided to block AMPARs and record NMDAR-mediated EPSCs to isolate pure monosynaptic L2/3 inputs to L5.

In order to determine whether the strength of the afferent pathway was affected by LPHN3, we had to control for variability in the density of electroporated neurons between mice since the number of ChR2-expressing neurons directly contributes to the number of light-sensitive synapses and therefore the strength of light-evoked L2/3 inputs to L5. When field potentials were recorded with an electrode positioned adjacent to the whole cell pipette (Figure 1C,D), we observed a small, fast signal time-locked to the light stimulus (Figure 1E). In the presence of tetrodotoxin (TTX) to block action potentials, the amplitude of this axonal field potential (AfP) was reduced by approximately 50%, while the EPSC was completely suppressed (Figure 1E). This indicates that our EPSCs are action potential-mediated and that a large fraction of the AfP is generated by axonal action potentials. We posit that current fluxed by ChR2 itself generates the TTX-insensitive portion of the AfP. Since both the fiber volley and ChR2 current are related to the local number of ChR2-expressing axons, and the AfP amplitude seemed to increase with subjective YFP fluorescence intensity, we decided to use the AfP as a measurement of the number of axons stimulated.

We found that light stimulation evoked AfPs and EPSCs of a wide range of amplitudes, but that EPSC amplitude scaled with AfP amplitude (Figure 1F). When we compared the amplitudes of EPSCs across conditions, we found that the amplitude of LPHN3 deficient inputs was reduced by 39% relative to control inputs, and that this effect was fully rescued by co-electroporation of shRNA-resistant LPHN3 (Figure 1G). For these same inputs, the mean AfP did not differ by condition (Figure 1H), indicating that the number of ChR2-expressing axons stimulated was not systematically different between conditions. The AfP and EPSC amplitudes were correlated and the slope of this relationship was 41% smaller in the shLPHN3 condition relative to Control and Rescue (Figure 1I). This reduced input-output relationship indicates that the L2/3-L5 microcircuit is weakened when L2/3 neurons lack LPHN3. Loss of LPHN3 could lead to two different kinds of defects that would weaken L2/3 input to L5: (1) reduced synaptic probability of release; or (2) lower axonal synapse density.

### LPHN3 does not affect probability of release at L2/3-L5 synapses

**Figure 2:**
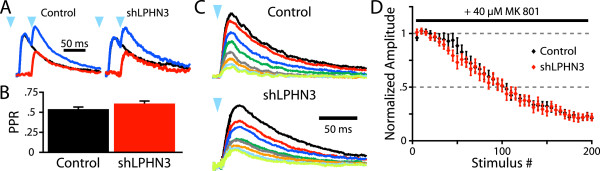
**LPHN3 does not modulate probability of release at L2/3-L5 synapses. (A)** Example average light-evoked NMDAR EPSCs recorded at +40 mV for Control and shLPHN3 electroporation conditions. Black traces are responses to a single light flash (blue arrowhead), blue traces are responses to two flashes at 20 Hz, and red traces are the subtraction. The PPR was then calculated as the ratio of the peak amplitudes of red and black traces. **(B)** The PPRs of L2/3 inputs did not differ between conditions (t-test, t(68) = 1.896, *P* = 0.06. Control, 0.54 ± 0.03, n = 36; shLPHN3, 0.62 ± 0.04 n = 34). **(C)** Example average NMDAR EPSCs of consecutive sets of 25 sweeps recorded in the presence 40 μM MK 801. **(D)** Plot of normalized NMDAR EPSC amplitude by stimulus number for Control and shLPHN3 inputs. The mean number stimuli for the amplitude to reach 50% of baseline did not differ between Control (98.5 ± 8.4, n = 10) and shLPHN3 (89.6 ± 5.9, n = 11) conditions (t(19) = 0.88, *P* = 0.39). All descriptive statistics and graphs are mean ± SEM.

In order to see whether LPHN3 affects presynaptic function, we began by measuring the paired pulse ratio (PPR) of light-evoked L2/3 inputs to L5. Since the slow decay of the NMDAR-mediated EPSC leads to summation of synaptic currents within the timescale of short-term plasticity, we averaged responses to a single stimulus or 20 Hz stimulus pairs and subtracted the former from the latter to get a pure ‘second’ EPSC (Figure 2A). The PPR was then calculated from the subtracted trace and the average single EPSC. Using this paradigm, we found that shLPHN3 did not affect the PPR at L2/3 inputs to L5 pyramidal neurons (Figure 2B).

To more directly examine whether LPHN3 affects probability of release, we recorded NMDAR EPSCs in the presence of MK801, an NMDAR antagonist that irreversibly blocks open NMDAR channels [14,15]. When a stimulus is delivered, only NMDARs at active synapses are blocked and the bulk EPSC becomes progressively smaller with each stimulus. In this way, synapses with a high probability of release will be suppressed more quickly than those with a low probability of release. The rate of MK801 block at LPHN3-deficient inputs did not differ from control inputs, indicating that the probability of release was not affected by the loss of LPHN3 (Figure 2C,D). Together these experiments suggest that LPHN3 is not involved in modulating presynaptic function at synapses formed by L2/3 axons in L5.

### LPHN3 controls the number of synapses formed by L2/3 axons

**Figure 3:**
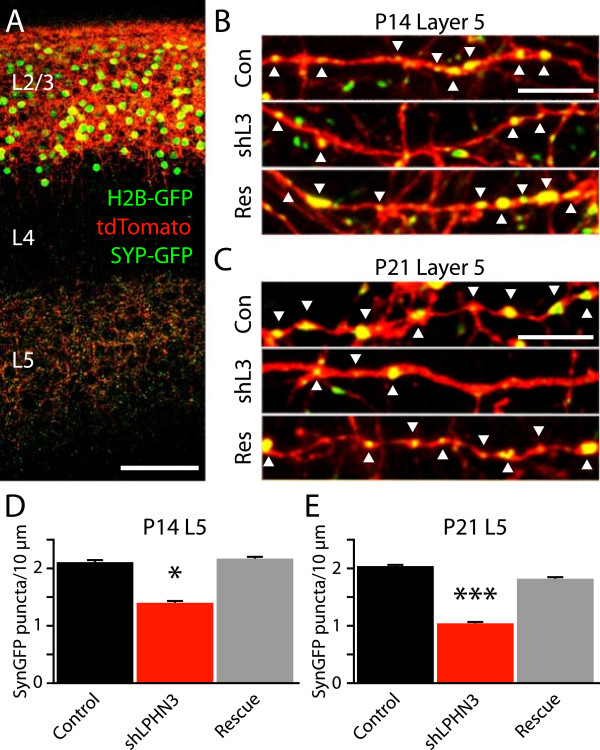
**LPHN3 controls the density of synapses formed by L2/3 axons in L5. (A)** Confocal image of GFP and tdTomato in a slice of somatosensory cortex from a P21 mouse electroporated *in utero* to express H2b-GFP, myrTdTomato, and Synaptophysin-GFP. Scale bar = 100 μm. **(B, C)** Example confocal images of tdTomato-positive axons in L5 decorated with Syp-GFP puncta (white arrowheads). Scale bar = 5 μm. **(D, E)** shLPHN3 axons, but not Rescue axons, have fewer Syp-GFP puncta than controls at both P14 (One-way ANOVA, F(2,104) = 4.49, *P* = 0.01: Control, 2.1 ± 0.2 puncta per 10 μm, n = 37; shLPHN3, 1.4 ± 0.2, n = 32; Rescue, 2.2 ± 0.2, n = 38) and P21 (One-way ANOVA, F(2,117) = 10.13, *P* <0.0001: Control, 2.0 ± 0.1, n = 40; shLPHN3, 1.0 ± 0.1, n = 37; Rescue, 1.8 ± 0.2, n = 43).

To determine whether LPHN3 affects the number of synapses formed by L2/3 axons, we electroporated L2/3 neurons with plasmids to express a set of fluorescent reporters; tdTomato to fill the cells, and Histone 2b-GFP and Synaptophysin-GFP (Syp-GFP) to mark nuclei and presynaptic terminals, respectively [16]. GFP-positive nuclei were apparent in L2/3, tdTomato-filled axons and dendrites were abundant in L2/3, unbranched axons traversed through L4 radially, and axons ramified extensively in L5 (Figure 3A). At high magnification, Syp-GFP puncta could be seen decorating tdTomato axons, often localizing to axonal varicosities (Figure 3B,C). When we counted Syp-GFP puncta along tdTomato axons, we found that shLPHN3 reduced the density of synaptic puncta by 34% at P14 (Figure 3D) and 49% at P21 (Figure 3E), and co-electroporation with shRNA-resistant LPHN3 restored synaptic puncta density to control levels. Combined with our electrophysiology results (Figure 2), this experiment indicates that LPHN3 regulates the strength of L2/3 input to L5 and controls the number of synapses made by L2/3 axons in L5.

### The extracellular domain of LPHN3 consists of two globular domains separated by a semi-rigid glycosylated linker region

**Figure 4:**
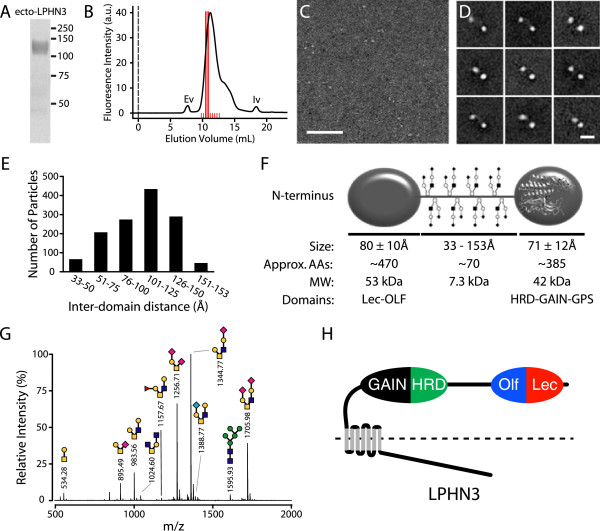
**The extracellular domain of LPHN3 consists of two globular domains separated by an O-glycosylated stalk. (A)** Coomassie blue stained gel of purified ecto-LPHN3 protein that was subjected to SEC in **(B)**. **(B)** SEC of the purified extracellular domain of LPHN3. The dashed line at volume 0 indicates the beginning of the injection. Fractions were collected between red lines. Fraction C3 was used for EM analysis. Ev and Iv indicate the excluded and included volumes, respectively. **(C)** Representative raw image of the extracellular domain of LPHN3. Scale bar is 100 nm. **(D)** Nine representative class averages of the individual particles. Scale bar is 20 nm. **(E)** Histogram of the distribution of inter-domain distances of individual particles. No particles were found to be separated by less than 33 Å or more than 153 Å. **(F)** Schematic model of the extracellular domain of LPHN3. Because of the slight difference is size of each domain, we tentatively assigned the larger domain to the Lectin and Olfactomedin domains, and the smaller domain to the HRD, GAIN, and GPS domains. In the proximal globular domain we manually superimposed a ribbon model of the available crystal structure [17]. **(G)** O-linked sugar analysis of the N-terminal domain of LPHN3 truncated at residue 571, just before the HRD. Several O-linked sugars and one N-linked glycosylation were present, as predicted by bioinformatics tools. Known glycans are indicated above the corresponding spectra. Sugars are represented as follows: yellow circle, galactose; blue square, N-acetylglucosamine; orange square, N-acetylgalactosamine; pink diamond, N-acetylneuraminic acid; red triangle, fucose; cyan diamond, N-glycolylneuraminic acid. **(H)** Domain organization of LPHN3. The extracellular domain of LPHN3 is composed of two globular regions separated by a flexible linker. The distal globular domain contains Lectin and Olfactomedin homology domains, while the proximal globular domain contains the HRD and GAIN domains.

To determine the overall architecture of the extracellular domain of purified LPHN3, we used single particle negative stain electron microscopy. To ensure sample homogeneity for the EM data collection, the purified protein was fractionated by size exclusion chromatography (SEC) and only one fraction of the main peak was deposited on the EM grid (Figure 4A,B). From the raw images it is apparent that the ectodomain of LPHN3 is composed of two globular domains separated by a variable distance (Figure 4C,D). To improve the signal-to-noise ratio, raw images were subjected to several rounds of averaging. The class averages of the single particles invariably show two globular domains (Figure 4D). Interestingly, the two globular domains are separated by a semi-rigid domain of variable length, too thin to visualize by EM (Figure 4D). This domain is composed by a stretch of approximately 70 amino acids enriched with Ser/Thr consistent with an O-linked glycosylation domain. The distance between the two domains varies between approximately 33 and 153 Å but the majority of the particles had a separation of approximately 105 Å (Figure 4E). Although these different distances simply reflect the variability of how each particle was deposited and stained on the grid, they indicate that the globular domains are separated in solution. Moreover, because when fully extended the distance between two amino acids is approximately 3.6 Å, and 65 residues can bridge approximately 230 Å, our data indicate that some stable secondary structure is probably present in the linker. By measuring the size of the two globular domains we find that they are not identical (71 Å and 80 Å) (Figure 4F). Although at this resolution we cannot identify them with certainty, based on the length of their amino acid sequence, we speculate that the larger globular domain likely represents the Lectin/Olfactomedin domains (approximately 470 amino acids) whereas the smaller domain (approximately 385 amino acids) likely consists of the Hormone Receptor Domain (HRD), GPCR Autoproteolysis Inducing (GAIN) domain, and GPCR Proteolysis Site (GPS) (Figure 4H). This result is consistent with the size of the available crystal structure of the GAIN domain [17]. To ascertain the source of the rigidity of the stalk domain, we analyzed the presence of O-linked oligosaccharide by MALDI-TOF/TOF mass spectrometry of per-O-methylated O-glycan and we determined that the protein has numerous O-linked sugars (Figure 4G). Using a deletion mutant that lacks the potential O-linked glycosylation domain, we conducted a similar mass spectrometry glycan analysis we found that the single N-linked glycosylation site predicted on the Asn532 and several O-glycan species were undetectable or observed in trace amount (data not shown). Thus, we found strong evidence that the region comprised by amino acids 495 to 565 is rigidified by a large number of O-linked sugars and one N-linked oligosaccharide, as predicted by bioinformatics tools.

### FLRTs and TENs bind to different but overlapping parts of LPHN3

**Figure 5:**
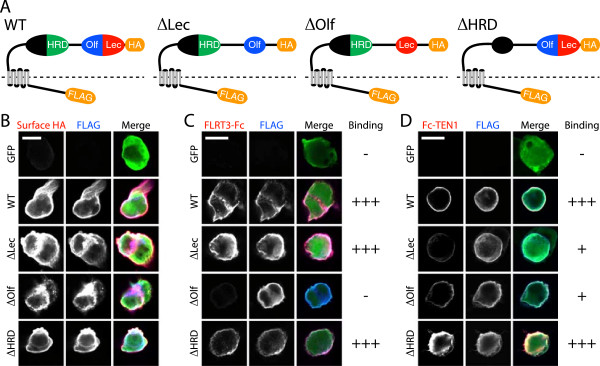
**The Olfactomedin domain of LPHN3 is required for FLRT3 binding while the Olfactomedin and Lectin domains both contribute to TEN1 binding. (A)** Schematics of epitope-tagged wild-type (WT) LPHN3 and protein-protein interaction domain mutants. The GPCR domain spans the plasma membrane (dotted line), the N-terminus of the extracellular domain has an HA tag, and the C-terminus of the intracellular domain has a FLAG tag. **(B)** Immunostaining for surface (HA live-labeling) and total (FLAG permeablized) WT and mutant LPHN3 proteins in transfected HEK cells. **(C)** Binding of ecto-FLRT3-Fc to HEK cells expressing GFP, WT LPHN3, or mutant LPHN3. **(D)** Binding of Fc-ecto-TEN1 conditioned media to HEK cells expressing GFP, WT LPHN3, or mutant LPHN3. Binding was categorized as strong (+++), weak (+), or absent (-). Scale bars = 10 μm.

LPHN3 has two classes of identified ligands, the FLRTs and TENs [8]. In order to map the ligand-binding domains of LPHN3, we created a series of deletion mutants lacking the identifiable conserved protein-protein interaction domains of the LPHN3 extracellular domain: Lectin, Olfactomedin, and Hormone Receptor (HRD) domains (Figure 5A). First we verified surface expression of all mutant LPHN3s by surface staining for an extracellular HA epitope tag (Figure 5B), and confirmed that control Fc protein did not bind to LPHN3 expressing cells (data not shown). Then we expressed LPHN3 constructs in HEK cells and assessed binding of soluble FLRT3 ectodomain (FLRT3-Fc) by immunofluorescence. We observed strong FLRT-Fc binding in wild-type, ΔLectin, and ΔHRD conditions, but deletion of the LPHN3 Olfactomedin domain (ΔOlfactomedin) nearly abolished FLRT3 binding (Figure 5C). Next we tested binding to the ectodomain of Teneurin-1 (Fc-TEN1), and found that both the ΔLectin and ΔOlfactomedin mutations compromised the ability of LPHN3 to bind Fc-TEN1 (Figure 5D). Finally, since an interaction between NRXNs and LPHN1 has recently be reported [18], we tested binding of NRXN1β(-S4)-Fc to LPHN3. Binding of NRXN1β(-S4)-Fc to LPHN3 was not reliably detected above background, whereas NRXN1β(-S4)-Fc bound strongly to LRRTM2-expressing HEK cells (data not shown). Thus we confirmed that FLRTs and TENs both bind to LPHN3, but that their binding involves different but partially overlapping portions of the LPHN3 extracellular domain that are constituents of the distal globular domain.

### The Olfactomedin domain is required for the synapse-promoting function of LPHN3

**Figure 6:**
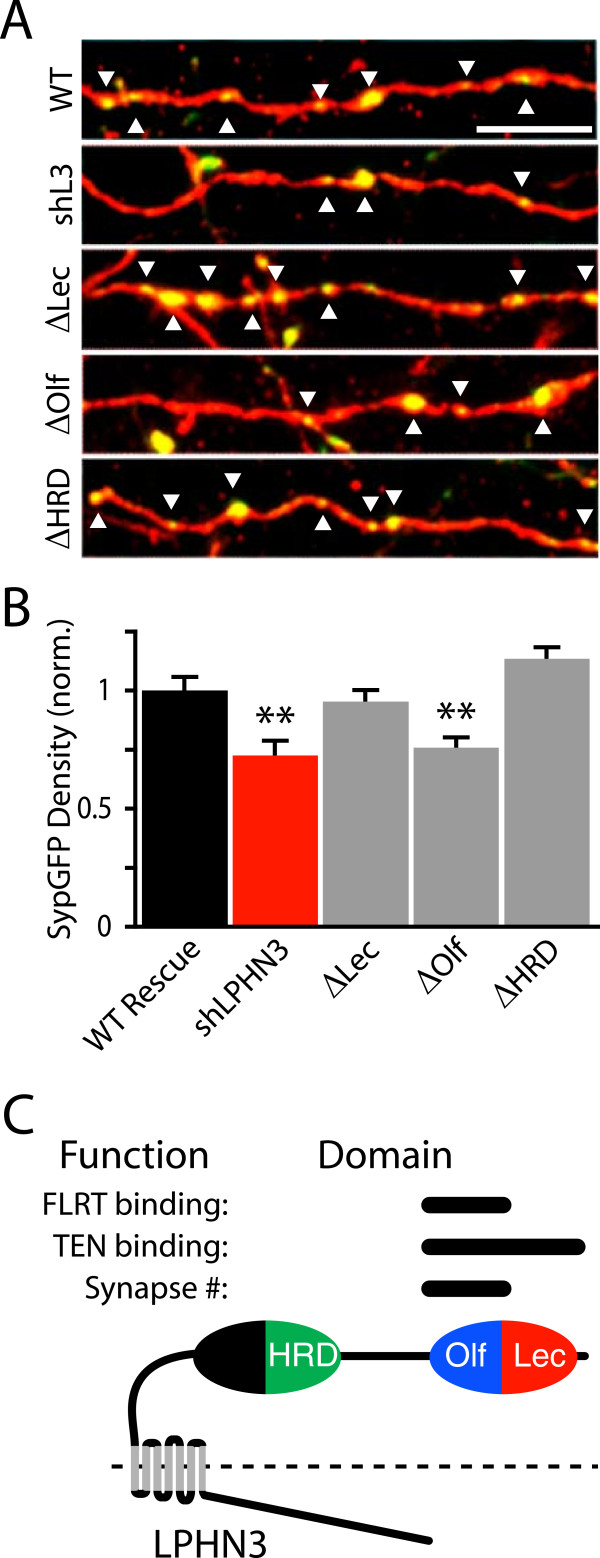
**The Olfactomedin domain of LPHN3 is required for development of normal synaptic output density. (A)** Example images of L2/3 axons in L5 from P14 mice electroporated with Syp-GFP shLPHN3 plasmids and wild-type (WT) or mutant LPHN3 plasmids. Scale bar = 5 μm. **(B)** Deleting domains of LPHN3 differentially affects the capability of the protein to rescue axonal synapse density (One-way ANOVA, F(4,299) = 10.07, *P* <0.0001: WT Rescue, 1.00 ± 0.06, n = 63; shLPHN3, 0.72 ± 0.06, n = 55; ΔLec 0.95 ± 0.05, n = 66; ΔOlf 0.76 ± 0.04, n = 63; ΔHRD 1.13 ± 0.05, n = 57). Post-hoc Dunnett tests show that the shLPHN3 and ΔOlf conditions differ from the WT Rescue (*P* <0.01). **(C)** Schematic of the domain organization of LPHN3 and summary of structure-function results. Conserved protein-protein interaction domains are labeled in the schematic, with the functions they subserve indicated by the bars above them. The extracellular domain of LPHN3 is composed of two globular regions separated by a flexible linker. The distal globular domain serves as a ligand-binding module, and FLRTs and TENs interact with different portions therein.

After mapping the domains of LPHN3 involved in binding to FLRT3 and TEN1, we decided to test whether the ligand-binding domains of LPHN3 are required for its effect on synapse development. To assess the function of LPHN3 mutants, we co-electroporated wild-type or mutant LPHN3 plasmids along with shLPHN3 and analyzed synaptic puncta in L5 (Figure 6A). We found that the different deletions had different effects on synaptic puncta density; deletion of the Olfactomedin domain prevented rescue of the synaptic phenotype, while the HRD and Lectin mutants remained capable of rescue (Figure 6B). Together with our binding data (Figure 5B,C), these data suggest that the same region of LPHN3 - the Olfactomedin domain - is required for both binding to FLRT3 and supporting normal synaptic output density, while deletion of the Lectin domain, which impairs binding to TEN1, does not affect synapse density.

## Discussion

We previously identified FLRT3 as an endogenous LPHN ligand, and identified a role for FLRT3 in the development of glutamatergic synapses [8]. In this study, we found support for the hypothesis that presynaptic LPHN3 regulates the strength of L2/3 input to L5 (Figure 1) by controlling the number of synapses formed by L2/3 axons (Figure 3). Using single particle electron microscopy, we determined that the extracellular domain of LPHN3 is organized into two globular domains separated by an elongated O-glycosylated linker region (Figure 4). We mapped the ligand-binding domains of LPHN3, and found that the Olfactomedin domain is required for FLRT3 binding, whereas the Lectin and Olfactomedin domains are both involved in binding to TEN1 (Figure 5). The distal globular domain of LPHN3 contains both the Lectin and Olfactomedin domains, suggesting it may serve as a ‘ligand-binding module’ on a stalk long enough to bridge the synaptic cleft. Mutant LPHN3 lacking the Olfactomedin domain was not able to support the synapse promoting function of LPHN3, while the Lectin and HRD domains were dispensable (Figure 6). That the Olfactomedin domain was required while the Lectin domain was not necessary suggests that LPHN3 may need the capability of interacting with FLRTs but not Teneurins for this function. These results are consistent with our hypothesis that a trans-synaptic interaction between LPHN3 with FLRT3 positively regulates synapse development.

While our experiments suggest that FLRTs and not Teneurins interact with LPHN3 to support synapse development, Teneurins also have described roles in synapse and circuit development in *Drosophila*[19,20] and mouse [21]. In these studies, Teneurins have been postulated to act through trans-synaptic homophilic binding, but the interaction of LPHNs with Teneurins may support a presently unknown LPHN function. Compared to FLRTs, Teneurins may act at other types of synapses, at a different time in development, or to fulfill a separate function.

Alternatively, it may be that FLRTs and TENs are expressed by different subpopulations of neurons within L5. L5 can be divided into sub-layers (L5a and L5b) with different patterns of connectivity and molecular determinants of synapse development [16,22]. Our experiments may most accurately represent L5b. Interestingly, *Tenm1* may be more highly expressed in superficial L5 and *Flrt3* in deeper L5. Further studies examining FLRT and TEN expression with markers for sublayer or projection target are needed to elucidate expression of these genes by more specific subpopulations of neurons.

More thorough characterization of Latrophilin, FLRT, and Teneurin expression by cell type and subcellular localization would be valuable to understand the full range of locations within the nervous system that Latrophilin interactions may occur. Our experiments did not support the hypothesis that LPHN3 regulates presynaptic vesicle release (Figure 2); it may be that the role of LPHN3 in controlling the rate of synaptic connectivity that we describe here is its primary role, but it could have different functions in different contexts.

Since LPHNs may interact with several ligands, it is possible that the functions of LPHNs are diverse and ligand-dependent. NRXNs set a precedent for such a hypothesis; NRXNs can participate in trans-synaptic interactions with neuroligins, LRRTMs, and CBLN-GluRδ complexes [23]. Further describing the distribution of these presynaptic proteins, the selectivity and exclusivity of their trans-synaptic interactions, and functional consequences of binding to different ligands remains imperative in order to understand the molecular logic underlying synapse development. The diversity of synaptic organizing proteins might allow the synapses formed between different classes of neurons to exhibit unique functional properties, such as the striking presynaptic facilitation conferred by postsynaptic ELFN1 at synapses formed onto certain classes of interneurons [24].

Our structural data also reveal some parallels to precedents set by NRXNs and Neuroligins. We found that the extracellular domain of LPHN3 has a characteristic architecture, with the Lectin and Olfactomedin domains and the HRD and GAIN domains forming two globular domains separated by a linker region that is rigidified by O-linked sugars, thus constituting a stalk reminiscent of other synaptic proteins such as the neuroligins and the neurexins [25]. This feature suggests that the ligand-binding module of LPHN3 may extend across the synaptic cleft, placing it in a position to interact with postsynaptic FLRTs.

In light of the association between LPHN3 and ADHD [2-4] and our evidence indicating that LPHN3 plays a role in the development of glutamatergic synapses in the cortex, it is possible that developmental synaptic abnormalities might underlie ADHD. Prevailing models of ADHD have focused on catecholamine modulation of prefrontal cortex due to the therapeutic efficacy of drugs targeting dopaminergic and noradrenergic pathways [26]. We speculate that an underlying defect in the development of glutamatergic synapses in the cortex - as might be caused by mutations in LPHN3 - could be involved in the circuit dysfunction that manifests as ADHD in addition to dysregulation of the neuromodulatory environment.

## Conclusions

Latrophilins are a small family of adhesion GPCRs whose role in the nervous system has only recently come under scrunity. An important role at synapses for LPHNs has been implied by two observations, however: (1) LPHN1 is a receptor for the black widow spider venom protein α-latrotoxin, which potently stimulates exocytosis; and (2) variants in the LPHN3 gene have been linked to ADHD in humans. We investigated the function of presynaptic LPHN3 in mouse cortex using complementary optogenetic and anatomical techniques, and found that loss of presynaptic LPHN3 from L2/3 pyramidal neurons reduced the strength of L2/3 synaptic input to L5 and reduced the density of synaptic puncta formed by axons of L2/3 neurons in L5. We then investigated the structure of the LPHN3 extracellular domain with respect to its ligand binding and synapse promoting functions. Using single particle electron microscopy, we found that the extracellular domain consists of a pair of globular domains separated by a glycosylated linker region. The distal globular domain serves as a ligand-binding module, containing the overlapping but differentiable regions that bind to FLRT3 and Teneurin 1. Mutations in the Olfactomedin domain that prevent FLRT3 binding also impair synapse density, while mutations in the Lectin domain that impair Teneurin 1 binding do not affect synapse density. We hypothesize that the interaction of presynaptic LPHN3 with postsynaptic FLRTs, but not Teneurins, supports synaptic development.

## Methods

### Plasmids

pCAG Cre and pCAG H2b-GFP-2A-myrTdTomato were provided by Corey Harwell and Arnold Kriegstein. pCAG DIO Syp-GFP was provided by Sebastian Espinosa and Michael Stryker. pAAV EF1-DIO-hChR2(ET/TC)-eYFP was provided by Karl Deisseroth. The LPHN3 shRNA was previously described [8] and targets nucleotides 958-977 of mouse LPHN3 mRNA (NM198702.2). The LPHN3 rescue construct was generated by making the following silent mutations in the shRNA target sequence: CCgGAcGCtTAcAAaATcA. Deletions were made by PCR, removing the following nucleotides: ΔLec, 844-1,086; ΔOlf, 1,129-1,896; ΔHRD, 2,200-2,394. The construct used for EM experiment (see below) encompassed amino acid residues 21 to 944.

### *In utero* electroporation

All experiments involving mice in this study were carried out in accordance with protocols approved by the IACUC at the University of California San Diego and were performed in accordance with institutional and federal guidelines.

Embryonic day 16 (E16) mouse embryos from timed pregnant CD1 mice (Charles River) were injected intraventricularly with DNA and 0.01% Fast Green and electroporated with 5 mm platinum-plated tweezertrodes (BTX/Harvard Apparatus) and a CUY21 electroporator (BEX) using the following parameters: 36 V, 5 75 ms pulses at 1 Hz. For Syp-GFP experiments, the ratio of plasmids was 1:6:3:5 pCAG Cre: pCAG DIO Syp-GFP: pCAG H2b-GFP-2A-myrTdTomato: pCAG HA-LPHN3-FLAG. For ChR2 experiments, the ratio of plasmids was 1:3:1.3 pCAG Cre: pAAV EF1α-DIO-hChR2(ET/TC)-eYFP: pCAG HA-LPHN3-FLAG.

### Electrophysiology

Acute coronal sections of somatosensory cortex of 300 μm were cut at approximately P14 in an artificial cerebrospinal fluid (ACSF) consisting of (in mM): 83 NaCl, 2.5 KCl, 1 NaH_2_PO_4_, 26.2 NaHCO_3_, 22 glucose, 72 sucrose, 0.5 CaCl_2_, 3.3 MgSO_4_. Slices recovered at 32°C for 1 h, and were then maintained at room temperature. Whole cell recordings were made from L5 pyramidal neurons at room temperature in ACSF consisting of (in mM): 119 NaCl, 2.5 KCl, 1 NaH_2_PO_4_, 26 NaHCO_3_, 2.5 CaCl_2_, 1.3 MgSO_4_, 11 glucose, 0.1 picrotoxin, and 0.02 DNQX. EPSCs were evoked with 1 ms flashes of blue light controlled by a SmartShutter (Sutter Instruments) and delivered through the 40× objective. Cells were selected for recording from near the center of the band of ChR2-expressing axons seen in L5. The internal solution contained (in mM): 130 Cs-methanesulfonate, 5 NaCl, 10 EGTA, 10 HEPES, 10 phosphocreatine, and 2 Mg-ATP, pH 7.3 with CsOH, 290 mOsm. Data were analyzed in Clampfit 10 (Molecular Devices) and statistical analyses performed with Prism 6 (GraphPad).

### Immunohistochemistry and confocal imaging

Mice were transcardially perfused at P14 or P21 with 4% PFA in PBS, the brains post-fixed overnight, and 150 μm coronal sections cut on a vibratome. Slices were blocked and permeabilized overnight in PBS with 3% BSA and 0.2 Triton X-100, then immunostained with 1:2,000 Goat anti-GFP (Abcam ab6673) and 1:500 Rabbit anti-RFP (MBL PM005).

Slices were imaged at 63× on a Leica SP5 confocal microscope, and stacks acquired with a Z-step size of 0.5 μm. Axon segment length was measured using the Simple Neurite Tracer plugin [27], the GFP signal thresholded, and the number of GFP puncta along the axon segment counted manually. All Syp-GFP experiments were performed in cohorts of two to three litters and one to three mice per litter, with each step of the experiment performed in parallel. Imaging thresholds were selected to achieve a consistent maximum puncta size by surveying the images in each cohort, and the threshold held for each cohort. Imaging and analysis was performed blind to condition. Each cohort included a control, and control data were consistent between cohorts. Statistical analyses were performed with Prism 6 (GraphPad).

### Protein binding experiments

FLRT3-Fc protein was produced as previously described [8]. The extracellular C-terminus of TEN1 was cloned in frame with human Fc to create an Fc-TEN1 plasmid. Purified Fc proteins (4 μg/mL) or conditioned media were added to transfected HEK cells, the cells were incubated at room temperature for 1 h, then washed, fixed, and immunostained.

### LPHN3 expression and purification

To purify soluble LPHN3, the medium containing the secreted extracellular domain of LPHN3 was passed over a protein A column (Captiv-A PriMab affinity resin by RepliGen), washed and eluted by 3CPro cleavage in TNED (50 mM Tris pH 8.0, 150 mM NaCl, 1 mM EDTA, 1 mM DTT) and 5 μM Leupeptin. The eluted LPHN3 protein was fractionated by size exclusion chromatography using a Superdex 200 10/300GL (GE Healthcare) column at 4°C and fraction C3 was used for EM experiments.

### Glycan analysis

Glycan analysis was performed by the UCSD Glycotechnology Core Resource. O-glycan from protein sample was liberated by reductive-beta elimination followed by per-O-methylation. The methylated O-glycans were dissolved in MeOH and mixed in 1:1 ratio with super-DHB matrix and spotted on MALDI plate. The mass spectra were acquired in positive mode and the plausible structure of the ions was extracted from the database using GlycoWorkbench software. The structures were proposed after comparing the mono-saccharide composition analysis, although variation in the orientation and linkages are possible.

### Specimen preparation and single particle electron microscopy

The purified extracellular domain of LPHN3, at a concentration of approximately 500 ng/mL, was prepared for EM using the conventional negative staining procedure [28]. Images were taken at room temperature at a magnification of 60,000× using a Philips CM12 electron microscope equipped with a digital camera. The SPIDER suite program was used for image processing [29]. Particles were windowed into 120 × 120 pixel images. Approximately 1,700 particles were subjected to several rounds of alignment, multivariate statistical analysis, classification, and image averaging. Images were finally divided in 50 classes, of which nine representative classes are shown (Figure 4D).

## Abbreviations

ACSF: Artificial cerebrospinal fluid; ADHD: Attention deficit hyperactivity disorder; AfP: Axonal field potential; AMPAR: α-amino-3-hydroxy-5-methyl-4-isoxazolepropionic acid receptor; CBLN: Cerebellin; ChR2: Channelrhodopsin 2; ELFN: Extracellular leucine-rich repeat and fibronectin containing protein; EM: Electron microscopy; EPSC: Excitatory post-synaptic current; FLRT: Fibronectin leucine rich transmembrane protein; GAIN: GPCR autoproteolysis inducing; GFP: Green fluorescent protein; GluR: Glutamate receptor; GPCR: G protein-coupled receptor; GPS: GPCR proteolysis site; HRD: Hormone receptor domain; LPHN: Latrophilin; MALDI-TOF: Matrix-assisted laser desorption/ionization - time of flight; NMDAR: N-methyl-D-aspartate receptor; NRXN: Neurexin; PPR: Paired pulse ratio; shRNA: Short hairpin ribonucleic acid; TEN: Teneurin; TTX: Tetrodotoxin; YFP: Yellow fluorescent protein.

## Competing interests

The authors declare that they have no competing interests.

## Authors’ contributions

MLO’S designed, performed, and analyzed confocal imaging and electrophysiology experiments and drafted the manuscript. FM and SvD performed single-particle electron microscopy experiments. AG and DC contributed to the conception, design, and coordination of the study. All authors participated in editing of the manuscript and approved the final manuscript.
